# Reply: Parkinson’s disease in GTP cyclohydrolase 1 mutation carriers

**DOI:** 10.1093/brain/awu309

**Published:** 2014-11-14

**Authors:** Niccolo E. Mencacci, Alan M. Pittman, Ioannis U. Isaias, John Hardy, Stephan Klebe, Kailash P. Bhatia, Nicholas W. Wood

**Affiliations:** 1 Department of Molecular Neuroscience, UCL Institute of Neurology, London WC1N 3BG, UK; 2 IRCCS Istituto Auxologico Italiano, Department of Neurology and Laboratory of Neuroscience – Department of Pathophysiology and Transplantation, ‘Dino Ferrari’ Centre, Universita` degli Studi di Milano, 20149 Milan, Italy; 3 Reta Lila Weston Institute of Neurological Studies, UCL Institute of Neurology, London WC1N 3BG, UK; 4 Department of Neurology, University Hospital, 97080 Würzburg, Germany; 5 Parkinson Institute, Istituti Clinici di Perfezionamento, 20126 Milan, Italy; 6 Sobell Department of Motor Neuroscience and Movement Disorders, UCL Institute of Neurology, London WC1N 3BG, UK

Sir,

Thank you for the opportunity to reply to the correspondence concerning our recent publication in *Brain*, ‘Parkinson’s disease in GTP cyclohydrolase 1 mutation carriers’ (Mencacci *et al.*, 2014). We read with great interest these letters and we thank the authors for their insights.

Guella *et al.* (2014) report the screening of *GCH1* in 528 Canadian cases with Parkinson’s disease and atypical parkinsonism and 290 matched controls. They identified two variants, the known pathogenic p.K224R (×2) and the novel variant p.A99D (likely pathogenic according to *in silico* prediction tools and interspecies conservation) in three unrelated cases with Parkinsons’s disease and the two benign variants p.P23L and p.P69L in one single control individual. The mutational frequency, excluding the aforementioned benign variants, was 0.56% (3/528) in cases versus 0% (0/290) in controls, consistent with the frequency we observed in our study (0.75% in cases versus 0.1% in controls).

This result is relevant as it represents the first independent confirmation that rare deleterious *GCH1* variants are enriched in patients with Parkinson’s disease compared to control subjects. Furthermore, they describe the post-mortem findings of one of the mutated patients, who presented at the age of 82 with DOPA-responsive asymmetric rest tremor. This showed a combination of brainstem Lewy body pathology together with the presence of tau-immunoreactive neurofibrillary tangles. To date, the only available brain pathology analysis of a *GCH1*-associated neurodegenerative parkinsonism case showed severe nigral neurodegeneration and Lewy bodies in surviving nigral cells and in the locus coeruleus (Gibb *et al.*, 1991; Segawa *et al.*, 2004). Further studies are needed to establish if the tauopathy described by Guella *et al.* (2014) in their case represents simply an incidental finding.

The finding that *GCH1* loss-of-function variants are not only responsible for childhood-onset DOPA-responsive dystonia, but are also associated with adult-onset neurodegenerative parkinsonism, is strengthened by the recent identification, through the meta-analysis of genome-wide association studies (GWAS) data deriving from ∼13 000 cases and 95 000 controls, that *GCH1* is also a low-risk susceptibility locus for Parkinson’s disease (Nalls *et al.*, 2014). This finding potentially extends the role of GTP cyclohydrolase 1 (GCH1) deficiency in the pathogenesis of Parkinson’s disease beyond carriers of rare deleterious coding mutations.

The causal link between GCH1 and Parkinson’s disease remains a matter of speculation. Ryan *et al.* (2014) expand the discussion of our manuscript and add insight into the possible pathogenic mechanisms that predispose *GCH1* loss-of-function mutation carriers to nigrostriatal degeneration.

In our paper we proposed various hypotheses whereby GCH1 and BH_4_ deficiency and consequent chronic reduction of dopamine levels may predispose carriers of *GCH1* mutations to nigral cell degeneration.

Ryan *et al.* (2014) point out that different cellular mechanisms secondary to BH_4_ deficiency, other than reduced dopamine levels, could contribute to the death of nigral dopaminergic neurons. BH_4_ acts as an antioxidant itself and is an essential cofactor for nitric oxide synthases (NOS) activity. Furthermore decreased BH_4_ levels have been demonstrated to lead to NOS uncoupling, which results in increased oxidative and nitrative stress (Chen *et al.*, 2014). The authors previously described that a haplotype of three SNPs (rs8007267, rs3783641 and rs10483639) at the *GCH1* genomic locus influences plasma GCH1 activity and BH_4_ levels (Antoniades *et al.*, 2008) and identified BH_4_ as a vascular defence mechanism against inflammation-induced endothelial dysfunction (Antoniades *et al.*, 2011). Consequently the authors propose that a link between BH_4_ levels, oxidative stress, and neuroinflammation could represent the mechanism underlying *GCH1-*associated Parkinson’s disease.

Fitting well with this model, and possibly supporting Ryan *et al.*’s view, we found that the *GCH1* SNP (rs11158026), recently identified as a risk variant for Parkinson’s disease (Nalls *et al.*, 2014), is in moderate linkage disequilibrium (r^2^ 0.457; D’ 0.932) with the SNPs constituting the functional haplotype. This possibly suggests a potential functional basis for the association of this variant to Parkinson’s disease.

The authors have also demonstrated the existence of an interaction between α-synuclein, mitochondrial function and GCH1 activity. Their work may support the compelling hypothesis that a pathogenic cascade occurs in nigral neurons, whereby increased levels of α-synuclein and mitochondrial dysfunction lead to decreased GCH1 activity and BH_4_ levels, which in turn may result in increased oxidative stress and cell death (Ryan *et al.*, 2014).

We believe that one of the outstanding questions is whether patients with DOPA-responsive dystonia eventually develop nigral neurodegeneration, or whether neurodegeneration can be avoided by dopaminergic replacement therapy. Answering this question will help to understand to what extent low dopamine levels play a role in nigral cell death, with obvious therapeutic implications for asymptomatic carriers of pathogenic variants.

Dopaminergic imaging studies performed in a few cases with classic DOPA-responsive dystonia (mostly genetically not confirmed) have shown no evidence of reduced nigrostriatal innervation (Snow *et al.*, 1993; Jeon *et al.*, 1998). This is consistent with post-mortem analysis of four extra cases showing normal nigral cell count (Furukawa *et al.*, 1999; Grotzsch *et al.*, 2002; Segawa *et al.*, 2013). However, Sawle *et al.* (1991) report that six cases with DOPA-responsive dystonia displayed modest but significant reduction in the uptake of ^18^F-fluorodopa into both caudates and putamen. Furthermore, Tadic *et al.* (2012) report that in DOPA-responsive dystonia cases parkinsonian signs are a relatively common residual motor sign following treatment, possibly suggesting underlying neurodegeneration.

With regards to this, the case report of Terbeek *et al.* (2014) is of great interest. They describe a 41-year-old patient carrying a known pathogenic *GCH1* variant (p.Y75S) with onset of classic DOPA-responsive dystonia at age 11. He was treated with l-DOPA (300 mg/day) from the age of 20 with good and sustained response. At age 41, because of rapid recurrence of dystonia after skipping a l-DOPA dose, dopaminergic imaging (^123^I-FP-CIT SPECT) was performed and showed severe bilateral and asymmetric reduction of putaminal tracer uptake, a pattern typical of idiopathic Parkinson’s disease. However, clinical examination, performed after withdrawing l-DOPA, revealed purely dystonic features without any obvious sign of parkinsonism.

In agreement with the interpretation of Terbeek *et al.* (2014), we believe that this case may indeed represent a case with overlapping DOPA-responsive dystonia and asymptomatic, as yet, nigrostriatal degeneration, possibly arguing against a neuroprotective role of dopamine replacement in *GCH1* mutation carriers.

Lastly, with regards to the letter by Furukawa and Kish (2014), we agree it is not easy to reconcile the evidence of nigral neurodegeneration that we and others have demonstrated in several individuals with *GCH1*-related parkinsonsim and the intact dopaminergic innervation showed in some other cases (Nygaard *et al.*, 1992; Kang *et al.*, 2004).

However, the phenotype of these latter cases, characterized by excellent and prolonged response to very small doses of l-DOPA and no motor fluctuations or dyskinesias in spite of decades of treatment, is very different from what we observed in the ‘neurodegenerative’ cases. It is therefore possible that there may exist two different types of adult-onset parkinsonism associated with *GCH1* mutations; on one side, a benign non-degenerative form, part of the phenotypic spectrum of metabolic GCH1-related striatal dopamine deficiency; on the other, a progressive form of parkinsonism with underlying nigral degeneration.

In conclusion, we anticipate that post-mortem analysis and longitudinal clinical, neuroimaging, and metabolic studies of larger series of *GCH1* mutation carriers—including asymptomatic carriers, individuals with classic DOPA-responsive dystonia and cases with adult-onset parkinsonism—will give way to important understandings of the pathogenesis of *GCH1*–associated Parkinson’s disease.

## Funding

This study was supported by the Wellcome Trust/Medical Research Council (MRC) Joint Call in Neurodegeneration award (WT089698) to the UK Parkinson’s Disease Consortium whose members are from the UCL/Institute of Neurology, the University of Sheffield, and the MRC Protein Phosphorylation Unit at the University of Dundee. This project was also supported by the National Institute for Health Research University College London Hospitals Biomedical Research Centre and the Grigioni Foundation for Parkinson Disease.
